# Reversible Splenial Lesion Syndrome Associated With Dengue Encephalopathy: A Case Report

**DOI:** 10.7759/cureus.48109

**Published:** 2023-11-01

**Authors:** Saishirini Yerremreddy, Neha Sai P Doddapaneni

**Affiliations:** 1 General Medicine, Dr. Pinnamaneni Siddhartha Institute of Medical Sciences and Research Foundation, Vijayawada, IND; 2 General Medicine, NRI Academy of Sciences, Guntur, IND

**Keywords:** dengue fever/complications, reversible splenial lesion syndrome, dengue encephalitis, dengue fever (df), dengue virus infection

## Abstract

Infection with the dengue virus can present with a variety of clinical manifestations that can range from asymptomatic or mild disease to severe hemorrhagic shock. In this report, we present a 25-year-old female patient with complaints of fever, headache, vomiting, and a reeling sensation for two days. On further examination, the workup for meningitis was negative, and the patient tested positive for dengue IgM antibodies. The MRI brain showed a restricted central lesion involving the splenium of the corpus callosum in favor of a cytotoxic lesion of the corpus callosum and a transient lesion of the splenium. Based on the MRI, a diagnosis of reversible splenial lesion syndrome (RESLES) was confirmed. Supportive treatment was initiated, and the patient made a complete recovery with no neurological deficits. A repeat MRI of the brain was done one month later, and it revealed complete resolution of the splenial lesion. If dengue fever is treated effectively, it frequently has a favorable prognosis with remission of uncommon, distinct radiological associations.

## Introduction

Dengue is among the most prevalent arboviral diseases and is the fastest-spreading tropical illness worldwide [1]. Dengue is prevalent in 128 countries, and more than 2.5 billion people globally are at risk of contracting the virus each year [1]. Dengue is a viral infection that is transmitted via *Aedes aegypti* mosquito bites to humans and is more prevalent in Asia, which accounts for 70% of the global disease burden [2]. According to the National Center for Vector Borne Disease Control, there were a total of 233,251 dengue cases and 303 fatalities reported in India in the year 2022 [3]. Infection with dengue can yield a wide range of clinical features, from mild fever to fatal hemorrhagic shock. A diverse range of neurological manifestations can be encountered, including encephalopathy, encephalitis, acute disseminated encephalomyelitis, Guillain-Barre syndrome, and neuro-ophthalmic involvement [4]. Due to the increase in the incidence of dengue fever worldwide, unusual manifestations of the disease are reported frequently. In this report, we discuss a patient with dengue encephalopathy with reversible splenial lesion syndrome (RESLES).

## Case presentation

A 25-year-old female patient came to the outpatient clinic in Andhra Pradesh, India, with complaints of fever, headache, vomiting, and reeling sensation for two days, followed by an altered sensorium for one day. She also reported having myalgia, but she did not have fits or any bleeding manifestations. The patient had a past medical history of sinusitis and has been on medication for the past two years. The patient did not have a history of travel in the past two months, and she is a non-smoker and non-alcoholic.

On examination, she was febrile with a temperature of 100.6 F and altered sensorium with a Glasgow coma scale (GCS) of 11/15. Palatal petechiae and a blanching rash were present. The patients had no signs of pallor, icterus, or lymphadenopathy. The patient’s vitals are as follows: pulse rate of 110 beats/min, BP of 110/80 mmHg with no postural hypotension, and respiratory rate of 12/min. General and other system examinations were non-significant. The patient was then admitted to the Department of General Medicine at the Hospital at Dr. Pinnamaneni Siddhartha Institute of Medical Sciences and Research Foundation, Vijayawada, India. Upon further examination, the patient had elevated platelets, alanine transaminase (ALT), and aspartate aminotransferase (AST) levels (Table 1).

**Table 1:** Lab investigations showing elevated platelet, ALT, and AST levels ALT: Alanine transaminase, AST: Aspartate aminotransferase

Parameter	Patient Value	Normal Value
Total WBC	5,000 cells/µl	4,500-11,000 cells/µl
Neutrophils	59%	40-60%
Hemoglobin	13.5 g/dL	13.5-16.5 g/dL
Platelet count	750,000/µl	150,000- 450,000/µl
Alanine aminotransferase (ALT)	160 U/L	< 50 U/L
Aspartate aminotransferase (AST)	75 U/L	< 50 U/L
Total bilirubin	0.5 mg/dl	0.1-1.2 mg/dl

The urine analysis and coagulation profile of the patient were normal. A lumbar puncture was performed, and the CSF was clear. The CSF analysis revealed only slightly elevated protein levels (Table 2). The CSF gram stain and bacterial culture were negative, ruling out meningitis.

**Table 2:** CSF analysis showing mildly elevated protein levels

CSF Parameters	Lab Value	Normal Value
Total WBC	3 cells/mm³ (Lymphocytes)	0-5 cells/mm³
RBC	0 cells/mm³	Nil
Protein	50 mg/dL	15-40 mg/dL
Glucose	80 mg/dL	50-80 mg/dL

**Figure 1:**
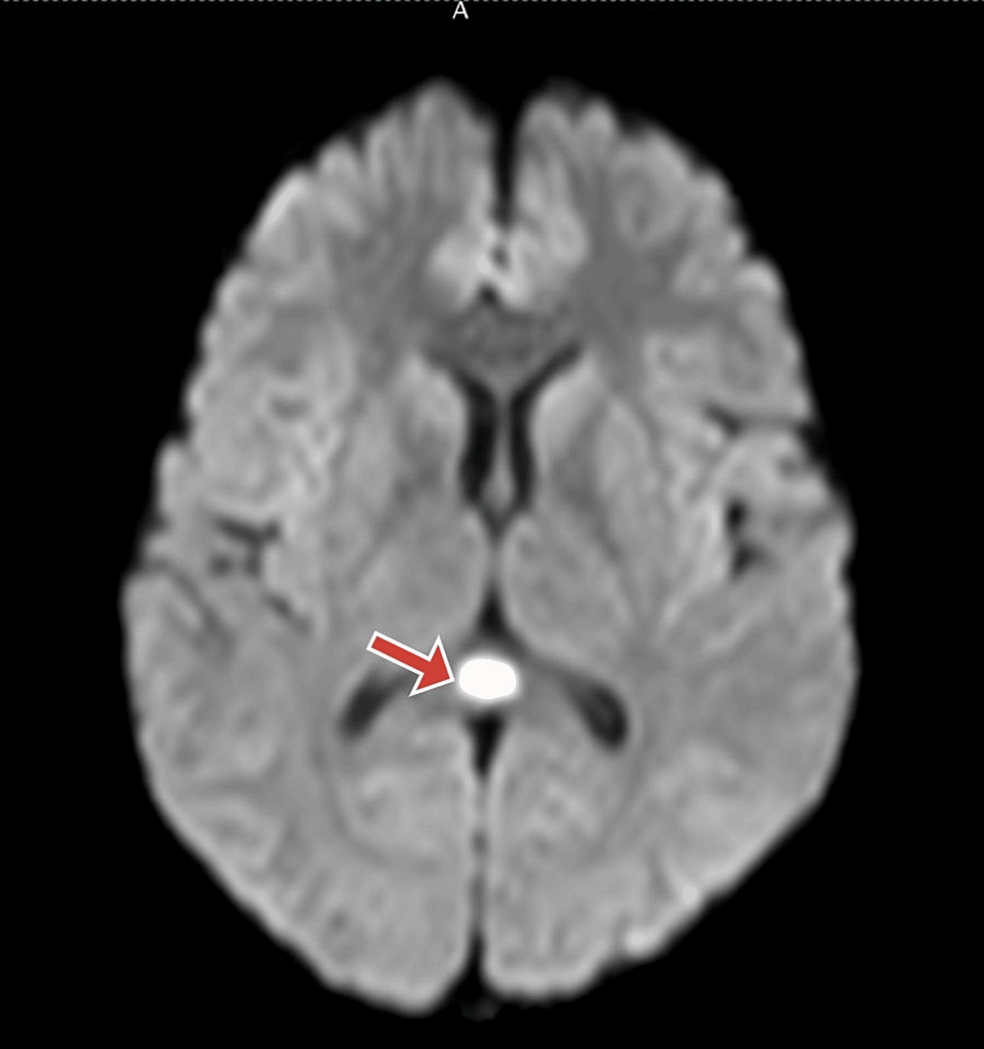
MRI of the brain The axial diffusion-weighted image shows a small central lesion involving the splenium of the corpus callosum with increased signal intensity.

**Figure 2:**
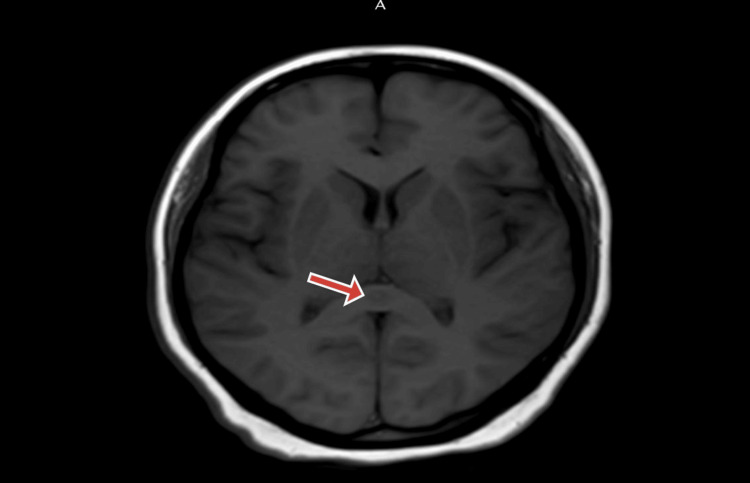
MRI brain axial FLAIR shows an oval area of increased signal intensity in the splenium corpus callosum FLAIR: Fluid-attenuated inversion recovery

On further testing, the dengue IgM antibody in the serum yielded a positive result. Blood and urine cultures did not show microbial growth. The patient was diagnosed with dengue encephalitis and was treated symptomatically with IV fluids and normal saline 100 ml/hour, injection of doxycycline 100 mg BD, and injection of paracetamol 1 gm IV BD. On further examination, an MRI brain showed a focally restricted central lesion involving the splenium of the corpus callosum in favor of a cytotoxic lesion of the corpus callosum and a transient lesion of the splenium (Figures 1-2). Based on the MRI brain, the diagnosis of RESLES was confirmed. Upon starting treatment, the patient achieved improvement to reach full recovery within eight days with no neurological deficits, and a follow-up MRI scan performed one month later, showed complete resolution of the splenial lesion.

## Discussion

Traditionally, dengue is an RNA virus that belongs to the *Flaviviridae* family and is considered a non-neurotropic virus that does not insinuate itself into the brain parenchyma or the meninges. The primary neurological presentation of altered sensorium with or without seizure along with a positive dengue antibody does not instantly confirm a diagnosis of dengue encephalitis. The widely used diagnostic criteria for dengue encephalitis include presentation of fever, signs of cerebral involvement such as altered consciousness with or without seizures, reactive IgM dengue antibody or NS1 antigen, and exclusion of other causes of encephalopathy [5,6]. Other causes of encephalopathy include hepatic encephalopathy, dyselectrolytemia, cerebral hypoperfusion, and cerebral edema [7]. However, numerous recent studies have sought to demonstrate potential radiological and clinical signs of true encephalitis.

One study has highlighted that transient splenial hyperintensities, also known as dot signs, are a feature of dengue encephalitis [8]. Transient splenial hyperintensities in dengue encephalopathy may be caused by an opening in the blood-brain barrier or due to osmotic and inflammatory damage resulting in intramyelinic edema or microvascular leak, among other factors [8,9]. Reversible splenial lesion syndrome is most likely attributed to cytotoxic edema except for high-altitude cerebral edema, in which vasogenic edema is thought to be the underlying mechanism. The reason for this propensity is that the splenium of the corpus callosum remains concealed, but one study has hypothesized that it is due to the absence of adrenergic tone [9]. Differentials must be ruled out, such as the absence of thalamic and hippocampal involvement and significant CSF pleocytosis, which are more likely to be classified as encephalopathy than encephalitis [7].

The signs and symptoms of the splenial lesion of the corpus callosum include confusion (50% to 60%), ataxia (33% to 43%), dysarthria (13% to 43%), seizure (10% to 40%), headache (16% to 23%), and hemiparesis (5% to 27%) [10]. The etiology of splenial lesions of the corpus callosum includes infectious viruses, i.e., influenza virus, rotavirus, measles, human herpesvirus 6 (HSV-6), adenovirus, mumps, Epstein-Barr virus (EBV), and *Escherichia coli*. Other causes include antiepileptic drug withdrawal, metabolic causes such as hypoglycemia and hypernatremia, high-altitude cerebral edema, and methylcobalamin deficiency [10,11].

In this case, the lesion in the splenium of the corpus callosum was found to be reversible. The hypothesis is again due to the reversibility of cerebral edema and the clearance of the virus. Lesions with an identified etiology have been documented to resolve within three to 21 days of onset [12]. The recognition of this uncommon and distinct radiological appearance in this patient with dengue fever gives reassurance of a favorable prognosis if the underlying condition is properly managed.

## Conclusions

In this case, RESLES was defined by the presenting characteristic of dengue fever complicated by encephalopathy. On further investigation, an MRI of the brain showed a small central lesion involving the splenium of the corpus callosum with increased signal intensity indicative of RESLES. After the initiation of treatment, the patient had a complete resolution of symptoms, along with a complete resolution of the splenial lesion, which was seen on a follow-up MRI one month later. Due to the increasing incidence of dengue worldwide, a wide range of atypical and unique radiological findings have been reported in association with the infection. The effective treatment of dengue fever often ensures a favorable prognosis and the remission of unusual and distinct radiological features.
